# The transoccipital parasigmoid canal of the vertebral artery through a vestige of the occipital vertebra

**DOI:** 10.1007/s00276-025-03568-1

**Published:** 2025-01-17

**Authors:** Mugurel Constantin Rusu, Corneliu Toader, Răzvan Costin Tudose

**Affiliations:** 1 Division of Anatomy, Faculty of Dentistry, “Carol Davila” University of Medicine and Pharmacy, Bucharest, 020021 Romania; 2 Division of Neurosurgery, Department 6– Clinical Neurosciences, Faculty of Medicine, “Carol Davila” University of Medicine and Pharmacy, Bucharest, RO-020021 Romania; 3 Clinic of Neurosurgery, “Dr. Bagdasar-Arseni” Emergency Clinical Hospital, Bucharest, RO-041915 Romania

**Keywords:** Occipital bone, Occipital condyle, Hypoglossal canal, Vertebrobasilar system, Atlas vertebra

## Abstract

**Purpose:**

The vertebral artery (VA) typically courses through the foramen magnum to enter the posterior cranial fossa. A transosseous course of the VA through the lateral part of the occipital bone is an unexpected finding. Such an anomaly of the VA is reported here.

**Methods:**

The archived angioCT file of an 81 y.o. female was studied on planar sections and by three-dimensional volume renderings.

**Results:**

The right VA looped above the transverse process of the atlas, and then it descended to enter a canal through the lateral part of the occipital bone. That canal was medial to the posterior condylar canal and the sigmoid sinus. It was termed the parasigmoid canal. The right VA entered the posterior fossa higher than the left VA, immediately posterior to the hypoglossal canal. A distinctive osseous plate was added on the inner side of the right occipital condyle and fused to the posterior arch of the atlas, narrowing the vertebral canal. A left osseous plate was fused medially to the occipital condyle. Both these plates may be remnants of an occipital vertebra. Thus, the right VA’s anomalous course may have resulted from an aberrant course of a hypoglossal artery near but not within the hypoglossal canal.

**Conclusion:**

The anomalous transoccipital course of the VA is a scarce but possible anatomical variation that may alter the neurosurgical landmarks, and it should be equally known by surgeons and interventionists.

**Supplementary Information:**

The online version contains supplementary material available at 10.1007/s00276-025-03568-1.

## Introduction

Different anatomic variations of the vertebral artery (VA) are known, such as abnormal origins, anomalies of the V2 transversary segment, anomalies of the V3 suboccipital segment, intracranial variations proximal to the origin of the basilar artery and anatomic variants of its branches [1, 2]. Bergman’s Encyclopedia of Human Anatomic Variation does not list a transosseous course of the VA through the occipital bone [1]. Here, we report a rare anatomic variation of a VA entering the posterior cranial fossa through a canal, not the foramen magnum.

## Material and method

The archived angioCT file of an 81 y.o. female was studied on planar sections and by three-dimensional volume renderings. The CT examination was performed on a 64-slice CT Somatom Definition As (Siemens), with a rotation time of 0.5 s, using a pitch of 1.2 and collimation of 1.2 mm. The technical parameters were detailed previously [3]. Anatomical variants were documented with Horos v3.3.6 software for macOS (Horos Project, Annapolis, MD, USA), on planar or curved planar sections, and via three-dimensional volumetric renderings. The principles of the Declaration of Helsinki were used to conduct the research. The Ethics Committee (affiliation #3) approved the study (approval no. 2093/1 March 2022). The transosseous canal of the VA through the lateral part of the occipital bone was found and documented anatomically.

## Results

**Fig. 1:**
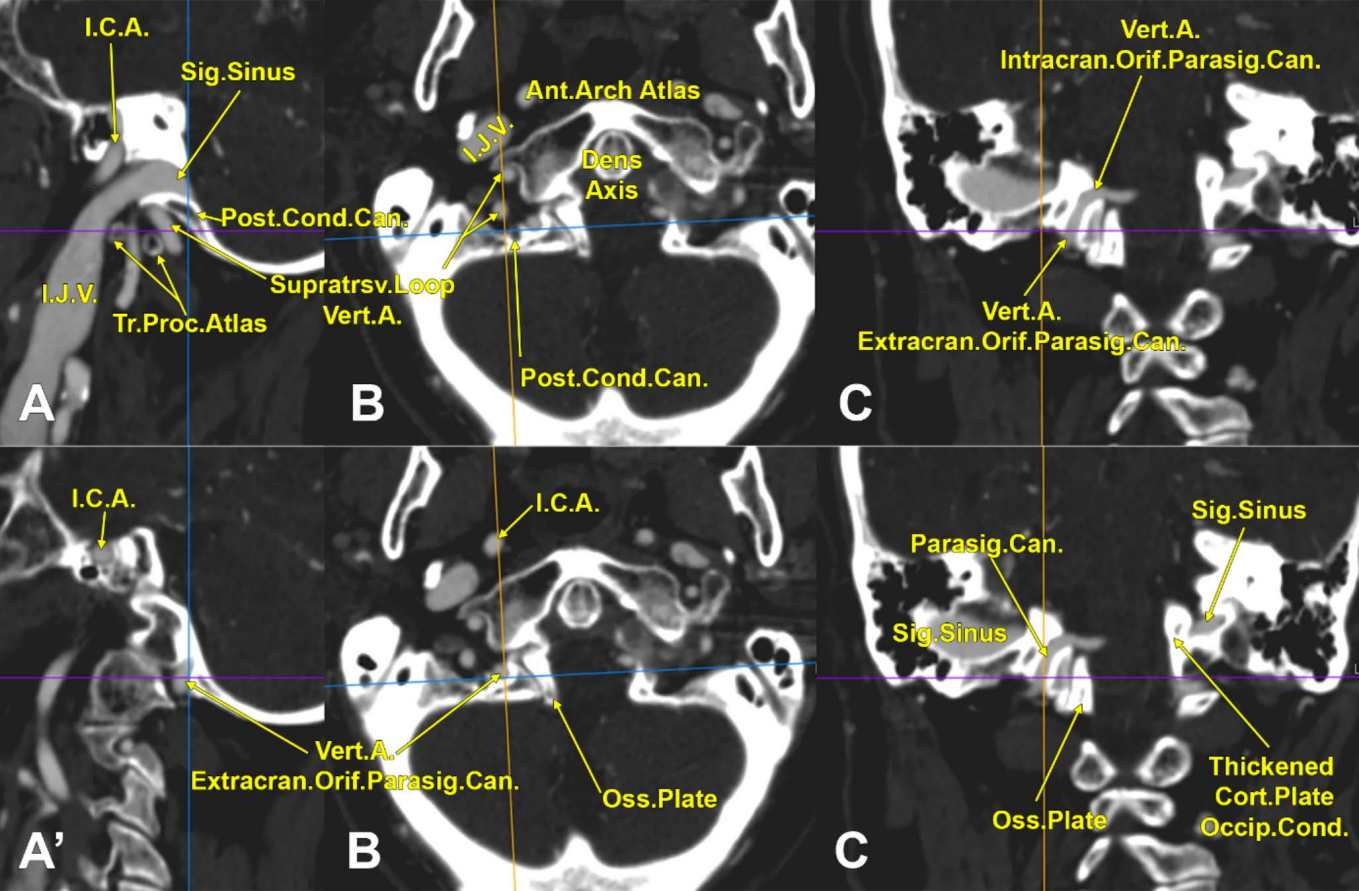
Two-dimensional, correlated sections (guidelines), right sagittal (**A**, **A’**, medial views), axial (**B**, inferior view) and coronal (**C**, anterior view) of the craniovertebral junction. The vertebral artery loops above the transverse process of the atlas and further courses through a parasigmoid canal to enter the posterior cranial fossa. An osseous plate narrows the right side of the vertebral canal. Ant., anterior; Cort., cortical; Extracran., extracranial; I.C.A., internal carotid artery; I.J.V., internal jugular vein; Intracran., intracranial; Occip.Cond., occipital condyle; Orif., orifice; Oss., osseous; Parasig.Can., parasigmoid canal; Post.Cond.Can., posterior condylar canal; Sig., sigmoid; Supratrsv., supratransversary; Tr.Proc., transverse process; Vert.A., vertebral artery

**Fig. 2:**
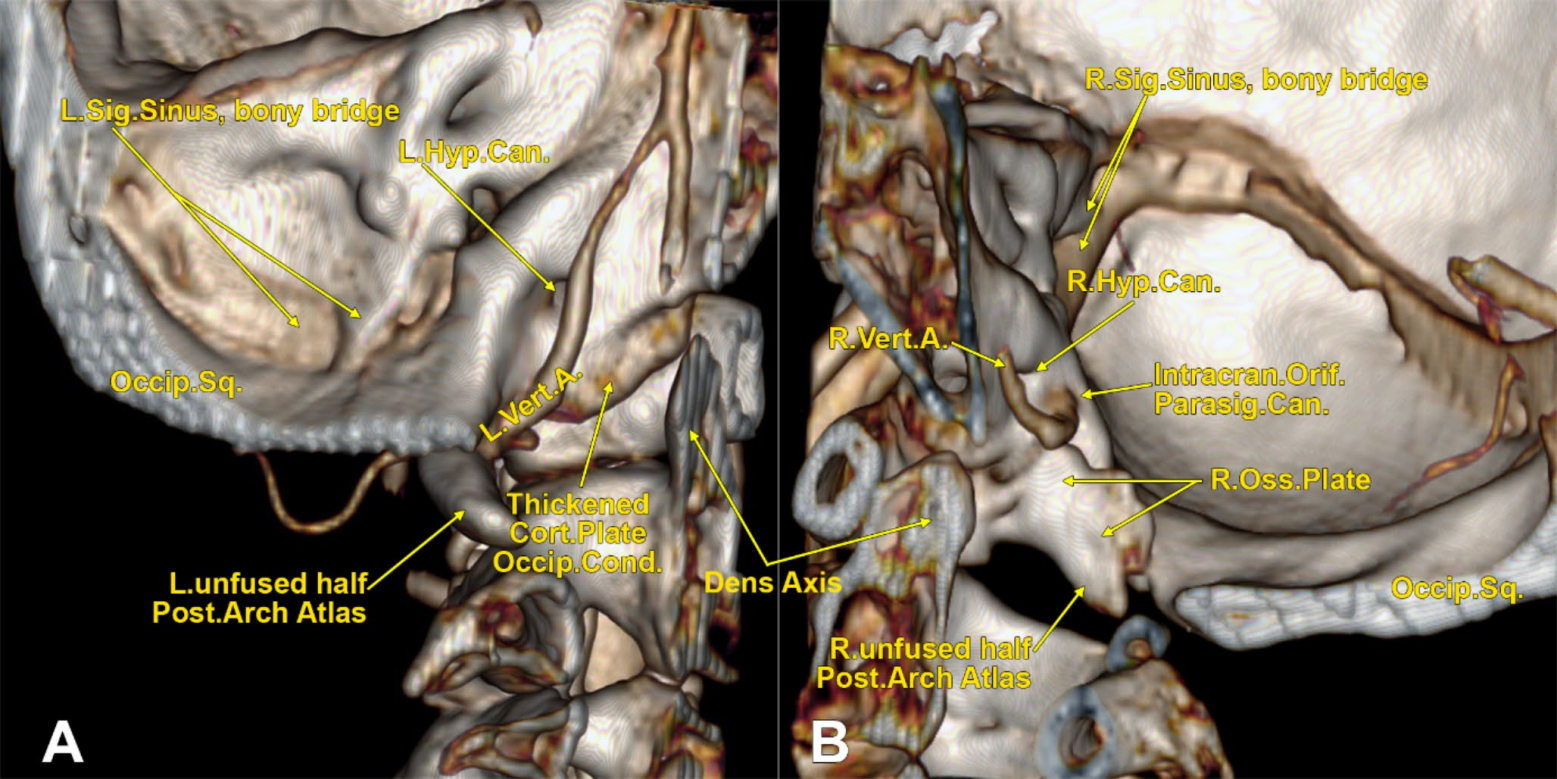
Three-dimensional volume renderings of the lateral sides of the foramen magnum. Internal views (**A**-left side, **B**-right side). Can., canal; Cond., condyle; Cort., cortical; Hyp., hypoglossal; Intracran., intracranial; L., left; Occip., occipital; Parasig., parasigmoid; Post., posterior; R., right; Sig., sigmoid; Sq., squama; Vert.A., vertebral artery;

A previously unknown anatomical variant of the VA was unilaterally found in an 83-year-old female case. It was observed that the right VA entered the posterior cranial fossa 12.8 mm higher than the left VA (Fig. 1C). The anatomical details of the right one were carefully gathered further (Figs. 1 and 2, Online Resource 1).

The right VA coursed through the transverse foramen of the atlas and continued upwards 6.45 mm to reach inferiorly to the sigmoid sinus sulcus, just before the sinus continued with the jugular bulb (Fig. 1A, B). Then, it turned downwards and coursed 12.21 mm to reach back the transverse process of the atlas at its posterior margin. It, therefore, described a supratransversary loop directed superiorly. Then, it redirected again and ascended 6.22 mm to the entrance of a transoccipital canal on the inner side of the extracranial opening of the posterior condylar canal and immediately posterior to the atlantooccipital joint (Fig. 1A**’**,** B’**). That unexpected canal the VA entered was regarded as a parasigmoid canal (PSC) because it was right above the root of the transverse process of the atlas and medial to the sigmoid sinus (Fig. 1C). The PSC was 12.78 mm long. The right VA entered the posterior cranial fossa at 7.91 mm postero-inferior to the inner opening of the hypoglossal canal (Fig. 2B). A distinctive osseous plate thick of 4.96 mm raised upwards from the right half of the posterior arch of the atlas and was found moulded at 1.07 mm on the inner side of the right lateral part of the occipital bone that, in turn, was traversed by the PSC and the posterior condylar canal (Figs. 1C and 2B). Therefore, the right osseous plate was narrowing the vertebral canal on the right side of the medullospinal junction (Fig. 1B, B**’**,** C**).

On the left side, a thickened cortical plate was observed on the inner side of the left occipital condyle (Fig. 1C). It was 3.79 mm thick; it did not narrow the vertebral canal or fuse inferiorly with the posterior arch of the atlas. That plate extended inferiorly on the medial side of the left atlantooccipital joint (Fig. 2A).

The posterior arch of the atlas was incomplete (Fig. 2). The occiput and atlas were not fused. The atlantooccipital joints were complete bilaterally, but the right occipital condyle was shorter (6.59 mm height) than the left occipital condyle (10.66 mm height). The heights of the lateral masses of the atlas were compensatory: the right was 14.07 mm high, while the left was 10.24 mm high.

A complete (left) or incomplete (right) bony plate bridged the sigmoid sinus on each side (Fig. 2).

## Discussion

A recent MR angiography study found a right VA penetrating the occipital bone lateral to the hypoglossal canal [10]. It was located on the infero-postero-medial bone of the right jugular foramen [10]. It, therefore, entered the posterior fossa at a higher level than the opposite VA [10]. Uchino and Suzuki (2024) explicitly listed the limitations of that study: CT images were not available, the bony canal of the VA was not clearly identified, and no detailed bony structures of the craniovertebral junction were observed [10]. However, they wrote that congenital skeletal variations, such as occipitalization of the atlas, were not observed [10]. Neither measurements were performed in that study. Therefore, the details we present here on such a transoccipital course of the VA are new.

In the case of Uchino and Suzuki (2024), the right VA ascended above the transverse process of the atlas and did not redirect towards the atlas, as we found in the present case. Therefore, the VA in Uchino and Suzuki’s case seemingly coursed through the jugular process’s paramastoid canal to reach the jugular foramen’s inner side and enter the posterior fossa. In our case, the right VA did not continue through a paramastoid canal to reach the jugular fossa’s floor. Still, it redirected back to the atlas by describing a supratransversary loop, entered the lateral part of the occipital bone immediately lateral to the condyle and coursed through a PSC to reach posterior and inferior to the endocranial opening of the hypoglossal canal. This is a different course, and our variant could not be superposed on Uchino and Suzuki’s one.

Uchino and Suzuki (2024) speculated that the anomalous right VA they found was formed by the persistence of one more cephalad primitive artery than the first intersegmental artery running in the spinal canal below C1, and neither it resulted from a persistent primitive hypoglossal artery because it did not pass through the hypoglossal canal [10]. Vertebral arteries running in the hypoglossal canal were reported long ago [4]. The embryologic hypothesis of Uchino and Suzuki (2024) also fits our results, but as we gathered osseous details, we’d instead reason differently.

In cases with an occipitalized atlas, the VA may traverse through the fused parts of the occipital and the atlas [5]. This does not fit our results because the occiput and atlas were not fused. Instead, we found additional bony plates bilaterally on the inner side of the lateral parts of the occipital bone. On the side of the anomalous VA course, the right one was fused inferiorly to the posterior arch of the atlas. The left one was fused superiorly with the occipital bone. These bony plates could be remnants of the proatlas– pieces of an occipital vertebra [6]. The proatlas contributes to the lateral masses and part of the posterior arch of the atlas [6]. This is not the first evidence of such bony plates added between the occipital condyle and the posterior arch of the atlas. Tominaga et al. (2002) reported an osseous process projecting from the occipital bone near the occipital condyle to the posterior arch of the atlas [9]. The typically coursing VA was completely occluded between that process and the posterior arch of the atlas while the patient’s head was turned to the opposite side [9]. The surgical decompression solved the rotational syndrome of the VA [9]. Tominaga et al. (2002) found that the osseous process was postero-lateral to the condyle and documented that it had not been reported previously [9]. An osseous plate on the inner side of the occipital condyle may narrow the foramen magnum and eventually compress the medulla or the medullospinal junction.

As reviewed by Muhleman et al. (2012), the structures created by variations of remnants of the proatlas include the paracondyloid processes and partial regressive occipital vertebrae [6]. The paracondyloid process is the rare Meckel’s paramastoid process on the inferior side of the jugular process of the occipital bone, lateral to the occipital condyle [7]. This may fit the anomalous VA of Uchino and Suzuki (2024) but not ours. Partial regression of the atlas creates enlarged occipital condyles with transverse processes [6]. Neither this possibility fits our finding nor excludes a proatlas provenance of the bony plates we found. Tominaga et al. (2002) also suggested that the osseous process they found may be a remnant of an occipital vertebra [9]. With this logic, the anomalous course of the right VA we found may be regarded as resulting from an aberrant course of a hypoglossal artery near but not within the hypoglossal canal.

During craniovertebral surgery, surgeons may need to drill the occipital condyle, attempting not to destabilise the atlantooccipital joint. The posterior condylar vein is the vascular landmark limiting the drilling. The hypoglossal nerve must be spared. When a transosseous course of the VA is not documented preoperatively, the artery may be severely injured during the surgical procedure, with a significant hemorrhagic risk, because it courses medially to the posterior condylar vein within the presumed safe drilling zone of the bone. A transcondylar neurosurgical route may risk the hypoglossal nerve, the condylar veins, and a transoccipital VA.

During far-lateral approaches of the cerebellomedullary cistern, the vagoaccessory triangle between the medulla, vagus, and accessory nerve is used [8]. It consists of a cranial suprahypoglossal triangle and an infrahypoglossal one, separated by the rootlets of the hypoglossal nerve [8]. When a transosseous VA exits postero-inferiorly to the entrance of the hypoglossal rootlets into the hypoglossal canal, such as in the present case, it would be an unexpected major vessel crossing the infrahypoglossal triangle to reach the ventral side of the medulla. Uchino and Suzuki’s variant of transosseous VA entered the posterior fossa above the foramen magnum [10], and in the absence of specific details, we can speculate it crossed the suprahypoglossal triangle. When a retrosigmoid approach is designed, the V3 segment of the VA must be protected by the use of various bony landmarks. A transosseous course of the V3 segment of the VA excludes it from the typical landmarks. When a V3 segment is not found, a transosseous course of the VA should be discriminated from an aplastic form of the VA.

In conclusion, the anomalous transoccipital course of the VA is a scarce but possible anatomical variation that may alter the neurosurgical landmarks, and it should be equally known by surgeons and interventionists.

## Electronic supplementary material

Below is the link to the electronic supplementary material.

Supplementary Material 1

## Data Availability

No datasets were generated or analysed during the current study.
